# Evidence of recent natural selection on the Southeast Asian deletion (--^SEA^) causing α-thalassemia in South China

**DOI:** 10.1186/1471-2148-13-63

**Published:** 2013-03-11

**Authors:** Qin-Wei Qiu, Dong-Dong Wu, Li-Hua Yu, Ti-Zhen Yan, Wen Zhang, Zhe-Tao Li, Yan-Hui Liu, Ya-Ping Zhang, Xiang-Min Xu

**Affiliations:** 1 Department of Medical Genetics, School of Basic Medical Sciences, Southern Medical University, Guangzhou, Guangdong, P.R. China; 2 State Key Laboratory of Genetic Resources and Evolution, Kunming Institute of Zoology, Chinese Academy of Sciences, Kunming, P.R. China; 3 Clinical Laboratory, Liuzhou Municipal Maternity and Child Healthcare Hospital, Liuzhou, Guangxi, 545001, P.R. China; 4 Key Laboratory of Animal Models and Human Disease Mechanisms of the Chinese Academy of Sciences & Yunnan Province, Kunming Institute of Zoology, Kunming, Yunnan, 650223, China; 5 Graduate School of the Chinese Academy of Sciences, Beijing, 100039, China

## Abstract

**Background:**

The Southeast Asian deletion (--^SEA^) is the most commonly observed mutation among diverse α-thalassemia alleles in Southeast Asia and South China. It is generally argued that mutation --^SEA^, like other variants causing hemoglobin disorders, is associated with protection against malaria that is endemic in these regions. However, little evidence has been provided to support this claim.

**Results:**

We first examined the genetic imprint of recent positive selection on the --^SEA^ allele and flanking sequences in the human α-globin cluster, covering a genomic region spanning ~410 kb, by genotyping 28 SNPs in a Chinese population consisting of 76 --^SEA^ heterozygotes and 138 normal individuals. The pattern of linkage disequilibrium (LD) and the long-range haplotype test revealed a signature of positive selection. The network of inferred haplotypes suggested a single origin of the --^SEA^ allele.

**Conclusions:**

Thus, our data support the hypothesis that the --^SEA^ allele has been subjected to recent balancing selection, triggered by malaria.

## Background

Malaria has long been known to be a prevalent selective pressure which has greatly shaped the human genome over the past 10,000 years [1,2]. The extensive overlap of the distribution of globin variants and the historical record for malaria prevalence suggests hemoglobinopathies might reflect a selective influence. Many other genes, such as glucose-6-phosphate dehydrogenase (*G6PD*), *ABO*, and human leukocyte antigen, are also believed to be evolutionarily interacted with malaria [2,3].

Strong natural selection can leave distinctive genetic imprint in DNA sequences, such as altering the levels of nucleotide variability and/or linkage disequilibrium. These signatures can be identified by comparison with the background distribution of genetic variation in humans, which is generally argued to evolve largely under neutrality [4]. These events have been studied since the advent of techniques for detecting positive selection in humans based on allele and/or haplotype frequency distribution pattern. Novel haplotype-based techniques have been widely used to demonstrate malaria-protective genes. For example, the limited number of observed haplotypes, low haplotype diversity, and extended haplotype homozygosity had indicated recent positive selection on G6PD in India [5]. The pattern of linkage disequilibrium suggests that the HbE mutation occurred recently in Southeast Asia [6]. In our previous study, we found that a 4 bp frameshift deletion of β-globin (β^CD41/42^), common in East and Southeast Asia, perhaps resulting from natural selection, spread widely with the help of gene conversion [7].

Alpha-thalassemia is one of the most common hemoglobinopathies. It is especially frequent in old-world malaria regions, such as Mediterranean countries, South-East Asia, Africa, the Middle East and in the Indian subcontinent [8]. It is caused by the genetic mutations which can lead to reduced production of α-globin chains, thus result in an imbalance between the proportions of α- and β-chains in hemoglobin synthesis. In normal individuals there are two copies of α-globin per chromosome. Most documented cases of α-thalassemia are caused by deletions involving one or both of the duplicated α globin genes, although increasing numbers of point mutations have been described. Therefore it is logical to divide α-thalassemia into two different types based on the number of remaining functional copies of the α-globin gene: α^+^-thalassemia (one copy is deleted or inactivated) and α^0^-thalassemia (both copies are deleted or inactivated). The more functional copies that are lost, the more severe the phenotype is presented: the loss of one copy of α-thalassaemia is phenotypically silent; the loss of two copies leads to mild anaemia; the loss of three copies leads to HbH disease that present anaemia of variable severity and the loss of all four copies is lethal.

Compared with α^+^-thalassemic deletions, α^0^ deletions are much less common on a global scale. Among the various α^0^ deletions, most of which are rare, the Southeast Asian deletion (--^SEA^) is remarkable for its high frequency. It is found most commonly in Southeast Asia, including Southern China. Our previous epidemiological surveys in Guangdong and Guangxi provinces found that the frequencies of --^SEA^ carriers were around 4-8% in Southern China [9,10]. Its localized distribution and relatively high frequency, considering the severity of its homozygous phenotype, might indicate a recent origin and selective advantage. However, no credible evidence of this has been reported to date. Some research suggests that --^SEA^ in the Thailand population had a single origin [11], but there is a lack of a detailed picture as to how selection elevated its gene frequency.

Fortunately, with the advent of dense maps of human genetic variation, it is now convenient to detect whether positive natural selection operates on variants of interest [12]. Here, we report the results of our fine-grained study of the --^SEA^ allele and haplotypes, based on a population from Southern China, to examine whether the --^SEA^ has been the target of recent natural selection.

## Results

### Allele frequency

**Figure 1:**
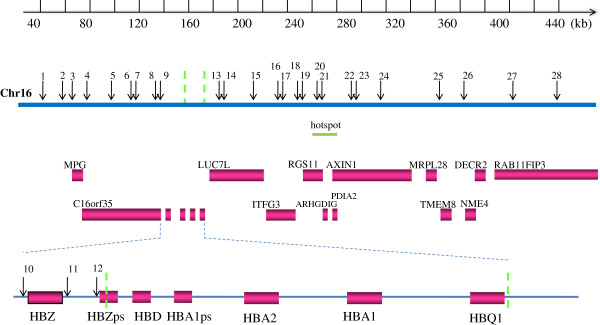
**Map of the 28 SNPs around the --**^**SEA **^**deletion.** Genes in the region are shown as boxes. SNPs are represented as arrows with numbers corresponding to Table 1. The dashed lines on chromosome 16 and the magnified view of the α-globin gene cluster indicate the breakpoints of the deletion. HBA2, hemoglobin alpha 2; HBA1, hemoglobin alpha 1; HBZps, hemoglobin zeta pseudogene; HBD, hemoglobin delta; HBA1ps, hemoglobin alpha 1 pseudogene; HBQ1, hemoglobin theta 1.

In this study, 28 SNPs encompassing the SEA deletion and covering a ~410kb region (Figure 1, Table 1) were genotyped in 214 samples from Guangdong and Guangxi of southern China. Information for all polymorphisms and the --^SEA^ allele in our samples are listed in Table 1. The derived allele frequency (DAF) for each SNP was computed in carriers and controls. Two SNPs, rs77308790 and rs3760053, which are non-common variants in the normal population (<0.1, Table 1) but were predominant in carriers.

**Table 1:** Profile of the 28 SNPs

				**Derived-allele frequency**		**P value for HWE test in CRS(N**^**c**^ **= 149)**
**No.**	**SNP ID**	**Position**^**a**^	**Polymorphism**^**b**^	**Carrier (N**^**c**^ **= 76)**	**Normal (N**^**c**^ **= 138)**	
1	rs2541593	43423	A/C	0.217	0.362	0.858
2	rs216606	57194	G/A	0.072	0.087	0.533
3	rs3785288	67230	T/C	0.138	0.261	0.849
4	rs2541622	76258	G/A	0.138	0.268	0.981
5	rs216091	97141	G/A	0.125	0.214	0.050^e^
6	rs2238368	110328	C/T	0.138	0.279	0.908
7	rs2562164	116743	G/A	0.289	0.529	0.112
8	rs2562185	132314	G/A	0.658	0.377	0.328
9	rs77308790^d^	135020	C/T	0.493	0.091	0.180
10	rs6600143	141389	A/C	0.132	0.264	0.605
11	rs2858925	148039	C/G	0.645	0.341	0.198
12	rs3760053^d^	151243	T/G	0.520	0.098	0.319
13	rs1211375	180281	C/A	0.757	0.482	0.774
14	rs3918352	187889	A/G	0.158	0.348	0.949
15	rs1203974	217459	A/G	0.704	0.464	0.541
16	rs11248914	233563	C/T	0.243	0.388	0.909
17	rs2252214	237751	C/T	0.171	0.341	0.662
18	rs4374177	246244	A/G	0.171	0.355	0.764
19	rs2239739	251854	A/G	0.730	0.457	0.918
20	rs9940585	262935	T/C	0.632	0.308	0.417
21	rs2685126	264053	G/A	0.211	0.319	0.967
22	rs214247	289222	T/C	0.066	0.163	0.166
23	rs1981492	296690	G/A	0.112	0.236	0.996
24	rs11648673	317795	G/A	0.053	0.127	0.155
25	rs1573733	363556	G/A	0.546	0.290	0.309
26	rs4984666	386363	A/C	0.224	0.420	0.409
27	rs1698232	425556	T/C	0.184	0.377	0.379
28	rs3785301	456923	G/T	0.717	0.551	0.426

^a^ Positions in chromosome 16 are based on NCBI, build 36.3.

^b^ Alleles on the positive strand; ancestral state is named first.

^c^ Number of individuals.

^d^ These two SNPs indicate a hitchhiking effect.

^e^ P < 0.05 in the Hardy-Weinberg equilibrium test.

### Extended LD around --^SEA^

**Figure 2:**
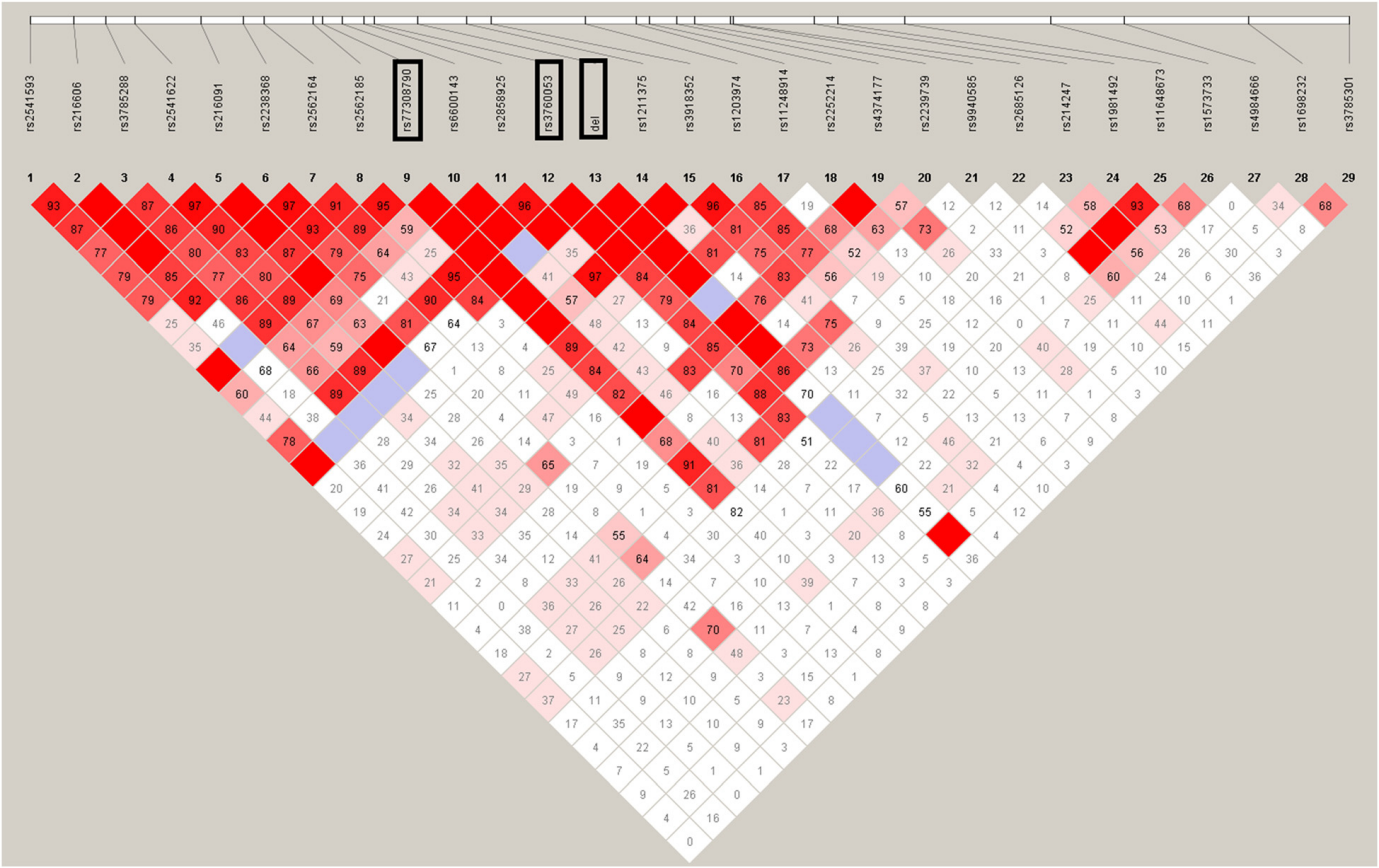
**LD structure constructed from 28 marker-inferred haplotypes in CRS dataset.** The three rectangles indicate the position of rs77308790, rs3760053 and the --^SEA^ allele respectively. The value in the square is the |D’| between the pair of loci. Darker red squares indicate higher values of |D’| with statistical significance (LOD > 2). Blue squares indicate high values of |D’| but with no statistically significant LD. White squares indicate low values of |D’| and LOD simultaneously.

**Figure 3:**
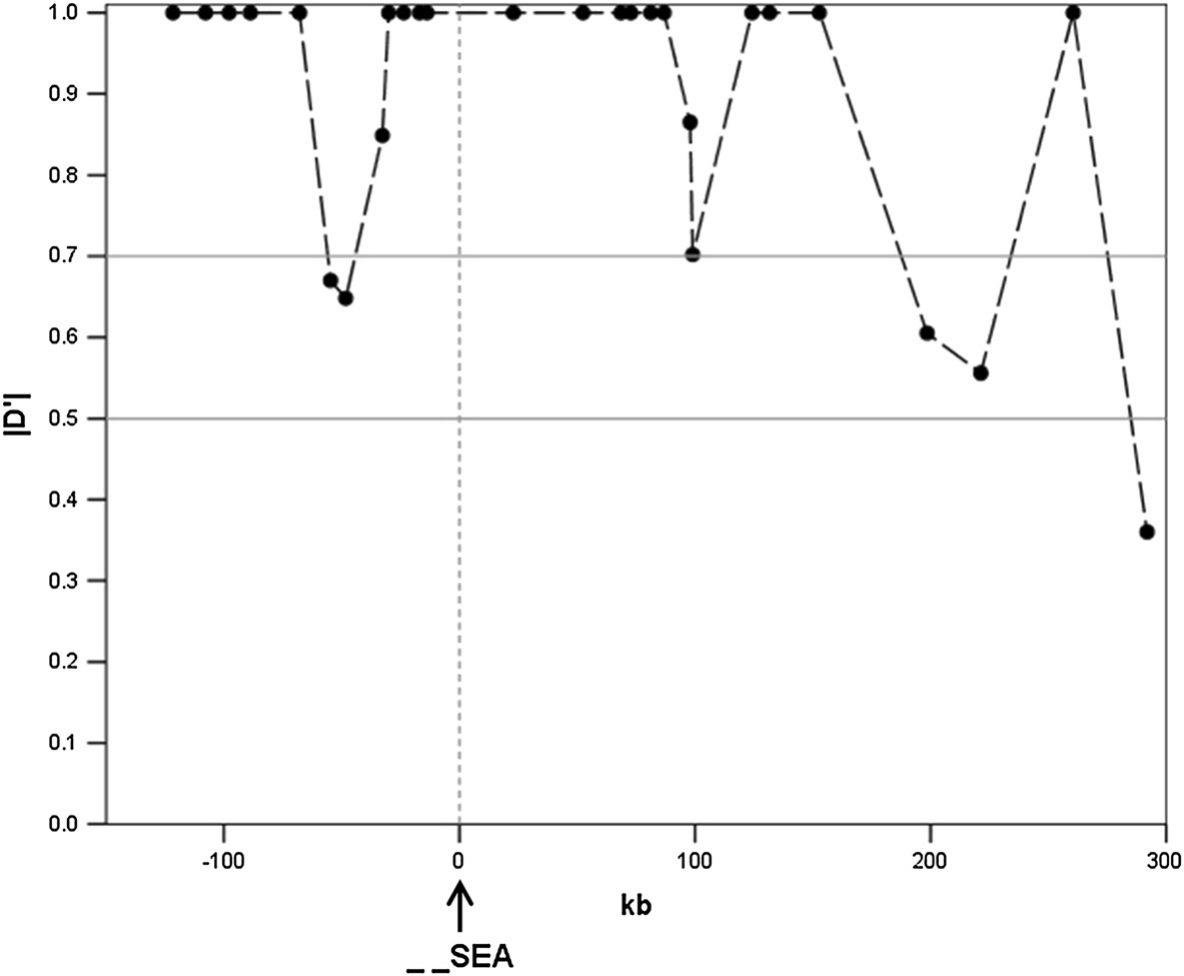
**Pair-wise |D’| between --**^**SEA **^**and other 28 biallelic markers.** The *x*- axis represents the relative position to the --^SEA^ allele (arrow at 0 kb) and the black dots indicate the distance and |D’| of corresponding markers.

We calculated |D’| [13] between markers to evaluate the LD pattern in the surveyed region for the constructed random sample (CRS; See Method). Pairwise comparisons show that strong LD within sites over short distance except those affected by recombination hotspot (Figure 2). However, while hotspot breaks down the LD downstream of the SEA deletion, the --^SEA^ still showed a high degree of LD with other SNPs (Figure 3), such as rs9940585, rs214247, rs1981492, rs11648673 and rs1698232 (although some of them with no statistical significance). Moreover, two SNPs, rs77308790 and rs3760053, showed a pattern similar to the --^SEA^ (Figure 2), indicating a hitchhiking effect due to linkage with the --^SEA^ allele.

### Great differences of recombination between haplotypes with the --^SEA^ and wild-type

The SEA deletion involves a large fragment, approximately 20.5 kb in length. The increased LD might be due to a lack of recombination in the deletion region on chromosomes carrying the causal allele rather than natural selection, especially when there is a hotspot located in the deletion region. To assess this, we scanned the genetic maps of CHS (Han Chinese in Singapore) and CHB (Han Chinese in Beijing). The local rate of recombination in deletion region were about 0.2 to 0.8 cM/Mb in these populations, which indicated that no hotspot was found in the deletion region compared with the average recombination rate of 1.2 cM/Mb in chromosome 16 [14]. Furthermore, we compared the minimum number of recombination events, namely RM [15] which was obtained using the four-gamete test, between haplotypes with the --^SEA^ allele and wild-type in our samples. The results of the two regions upstream and downstream of the deletion are provided in Table 2. It can be seen that recombination events seem to occur more actively on normal chromosomes than the --^SEA^ chromosomes in both regions.

**Table 2:** **Estimated the minimum number of recombination events RM for --**^**SEA**^**and normal chromosomes**

	**Upstream**	**Downstream**
--^SEA^	1	4
wild-type	8	13
difference	7 (>5*)	9 (>2*)

*95^th^ percentile in bootstrap approach.

### Test for recent selection on --^SEA^

**Figure 4:**
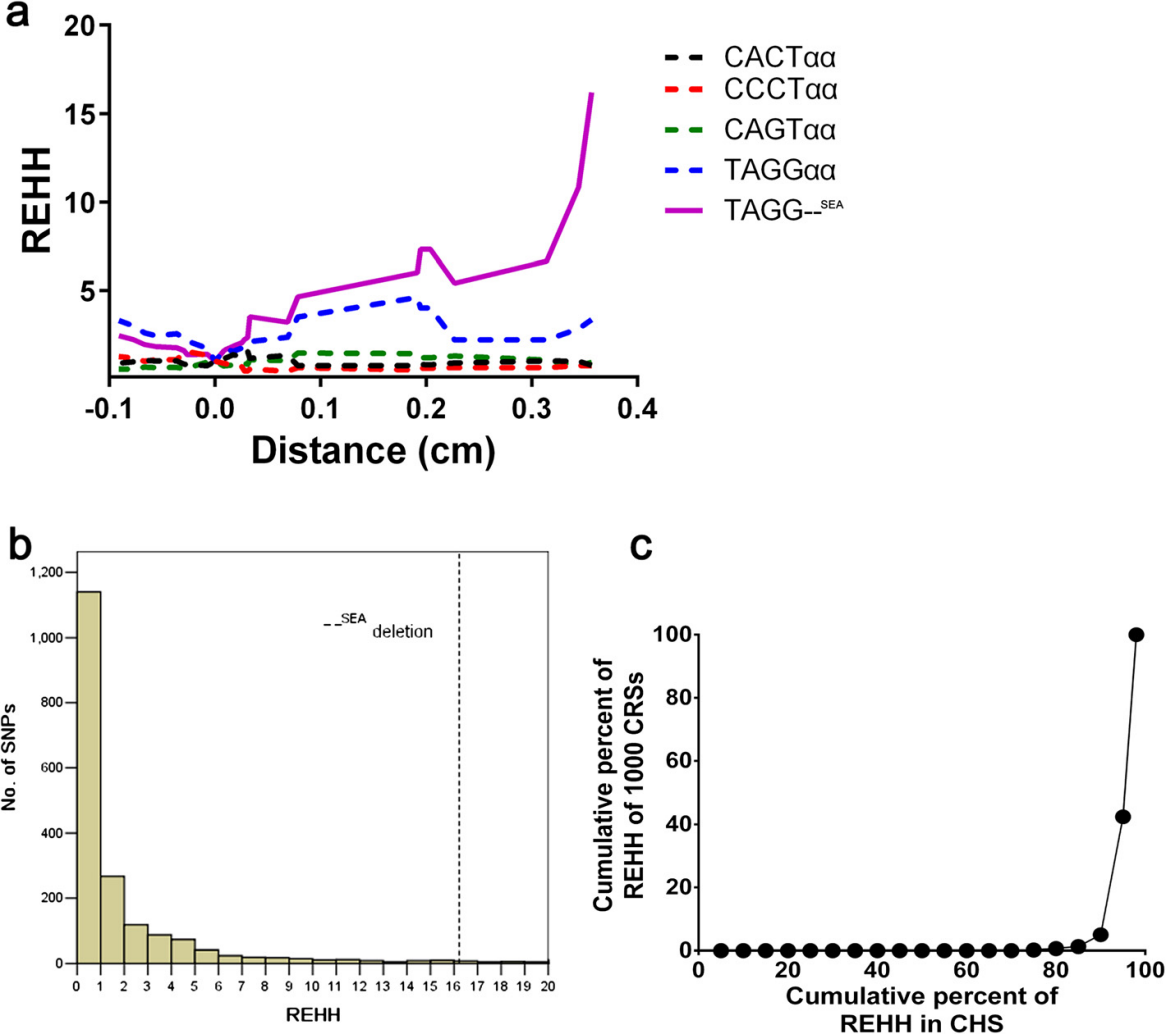
**Results of LRH test.** (**a**) REHH plots of the core region covering the --^SEA^ allele in CRS dataset. The values are plotted against the genetic distance from the selected core region. The plot of core haplotype containing --^SEA^ is indicated by the solid yellow line. (**b**) The empirical distribution of REHH value of SNPs at a distance of 0.35 cM on chromosome 16. Only those alleles with population frequency between 0.031 and 0.042 in the CHS were considered. The REHH value of --^SEA^ is indicated by the dashed line. (**c**) The plot of cumulative frequency of REHH value of 1000 constructed random samples vs. that of CHS. Most of the REHH values of 1000 CRS (~60%) were within the 5% extreme high value of REHH in CHS.

A long-range haplotype test (LRH) using the relative extended haplotype homozygosity (REHH) parameter was conducted to detect the signature of recent natural selection [16]. A variant under neutral evolution would take a long time to reach a high frequency and the LD around the variant would decay substantially during this period due to recombination. In contrast, alleles under positive selection will rise to a high frequency so quickly that long-range associations with neighboring polymorphisms are not disrupted by recombination. In this LRH test, we assigned the core region containing four SNPs, rs77308790, rs6600143, rs2858925, and rs3760053, and the --^SEA^ allele using the default parameters in the Sweep software (http://www.broadinstitute.org/mpg/sweep/). The REHH value of the deletion-bearing haplotype in CRS reached 16.22 on the centromere-proximal side at a distance of 0.35 centimorgans from the focal allele (Figure 4a). We obtained SNP data on chromosome 16 from the CHS population, which is close to Southern Chinese population, to calculate the empirical distributions of REHH values for core haplotypes. Only the core haplotypes with similar frequency of our data, i.e. 3.1% < MAF < 4.2%, were considered. Compared with other SNPs, the observed REHH values of the deletion haplotypes were significantly larger and the P-value was less than 0.044 (Figure 4b). To verify the reliability of the result, we regenerated 1000 CRS datasets to obtain a distribution of REHH for the LRH test. The result was consistent, with a mean value of 17.41, and most of the REHH values were within the 5% extreme high value (Figure 4c).

**Figure 5:**
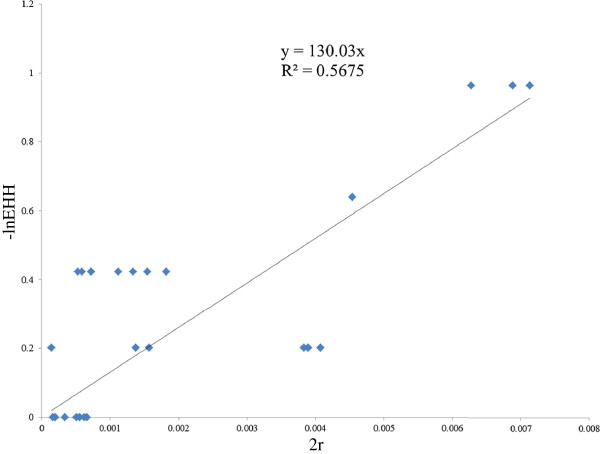
**Evaluation of the ages of the --**^**SEA **^**by linear regression of -ln(EHH) and 2r.** X-axis represents the 2*r, where r is the genetic distance between core and a given marker; Y-axis represents the –lnEHH of that marker. We could obtain vector of (r, EHH) for each marker and make a plot. Each diamond corresponds to a SNP.

An approach introduced by Voight [17] and coworkers was used to obtain a crude estimate of the age of the --^SEA^ variant. In this method, age was calculated using the equation, *Pr [Homoz] = e*^*-2rg*^, where *Pr [Homoz]* is the probability that two chromosomes are homozygous at a recombination distance *r* from the selected site, given a common ancestor *g* generations before the present. Here, we used a linear regression to evaluate the value of *g* through the transformation formula -ln(EHH) = g*2r based on our EHH data. As in Figure 5, the parameter of generation *g* was 130.03 in CRS. Assuming a generation time of 25 years for humans, the age is thus about 3250 years (If human generation time is 30 years, the age is 3909 years).

### Origin of the --^SEA^ allele

**Figure 6:**
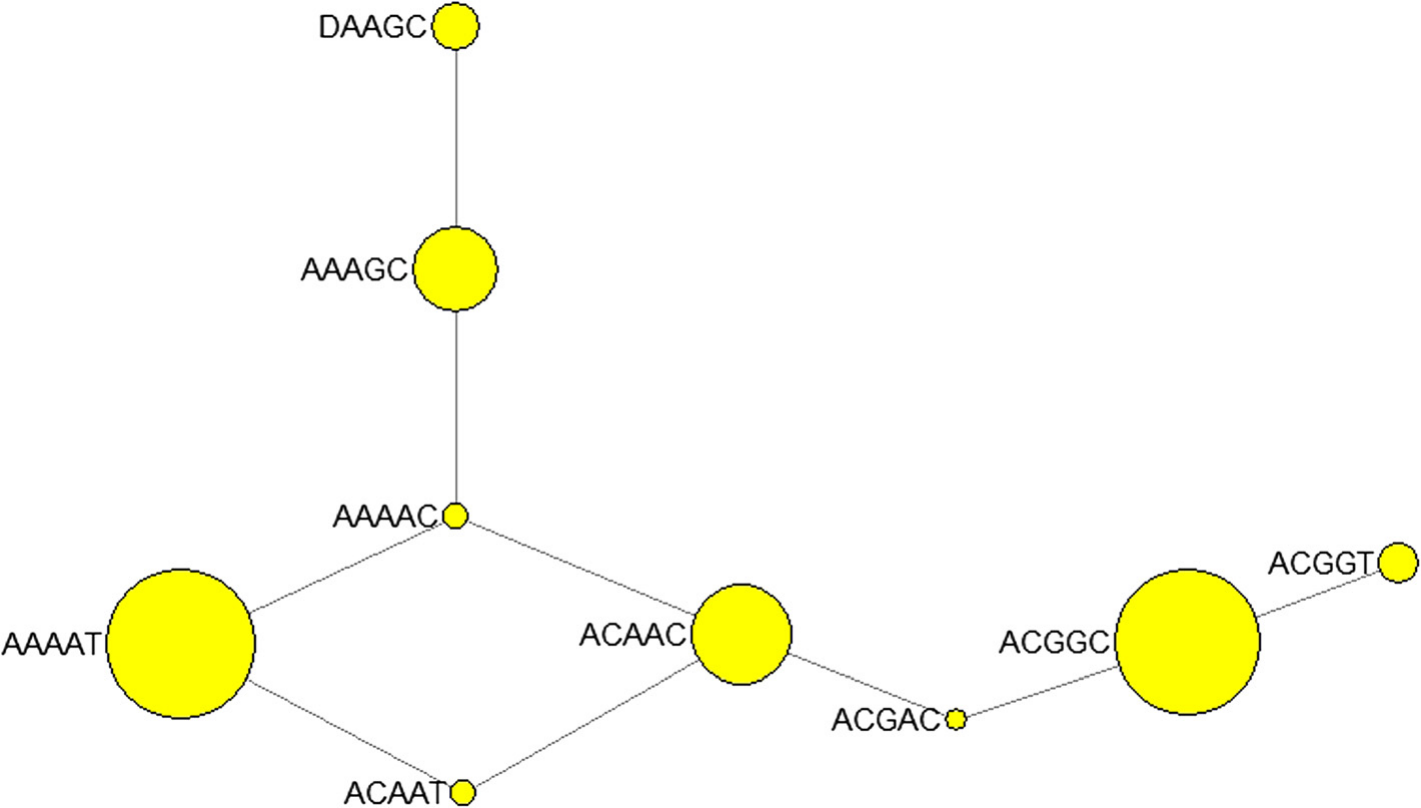
**Phylogenetic network of haplotypes of four SNPs and --**^**SEA **^**allele.** The four SNPs used to construct the network are rs1211375, rs3918352, rs1203974 and rs11248914. The first letter A in haplotypes is αα and D indicates --^SEA^ allele.

A network of haplotypes carrying the --^SEA^ and four SNPs adjacent to downstream of the breakpoint was constructed. There was only one haplotype carrying the --^SEA^ and it arose from the ααAAGC haplotype by a single mutation (Figure 6). The AAGC haplotype was common in our CRS population (15%) and prominent in East Asian populations in the HapMap project [18] but barely seen in the rest of the world, with a few exceptions such as CEU (only 2 in 300 chromosomes) and GIH (only 1 in 170 chromosomes).

## Discussion

Presumably the most common form of α^0^-thalassemia, --^SEA^, is maintained at a high frequency through heterozygote advantage by malarial selection. In malarious areas, carriers of this mutation will tend to have higher viability, leading to increased gene frequency until it is balanced by its loss in homozygotes who die before reaching sexual maturity. This process can elevate levels of LD around the target locus relative to that expected under a neutral model. From genotyping 28 SNPs around the SEA deletion, our results are consistent with this hypothesis. In the CRS population, mimicking the frequency of the variant in Southern China, extended LD was observed. In Asians, as well as African-Americans and European-Americans, strong LD is hardly observed for SNPs more distant than ~80 kb [6,19]; there is significantly extended LD from the --^SEA^ to SNPs outside the deletion locus (~200 kb), and the range would be much larger than the average range of strong LD expected in an Asian population.

Although it is difficult to evaluate the effects of large-scale fragment deletion on long-range LD accurately, this is unlikely to be the consequence of a lack of recombination in the deletion region on chromosomes. This is done by estimation of the minimum number of recombination events and an LRH test. First, the deletion region in the normal chromosome exhibits a low degree of recombination, which does not explain the difference between chromosomes carrying the --^SEA^ and the wild-type allele. Moreover, it is believed that recombination events in the deletion region only impact the correlation between upstream and downstream, but interaction of markers within upstream or downstream are not affected. However, we can see gaps between the --^SEA^ chromosomes and normal chromosomes in both regions. Another convincing argument is that the LRH test showed a significantly high REHH value, indicating selection favoring the --^SEA^ variant.

This signature probably results from the malarious environment through estimating the age of the --^SEA^ variant. Diseases that might be caused by parasites have been described in detail in early written records from 3000 to 300 BC [20]. By the beginning of the Christian era, malaria was widespread in Central, South, and Southeast Asia, including China [21]. Many variants conferring resistance to malaria appear to be recent polymorphisms from the last 5000–10000 years or less [1]. These records are consistent with our results, that the --^SEA^ variant is relatively young and arose within the last 3250 years. This estimate is crude and likely underestimates the true age of the alleles: First, this method assumes a star-like genealogy for the selected haplotypes; furthermore, the derived allele may be at an equilibrium stage for some time [22]. However, it is still believed that the SEA deletion is a very recent mutation, unlikely to have been present before the early migrations of populations into Asia. The haplotype ααAAGC from which the --^SEA^ arose was specific in Asian population, probably generated after modern humans migrated into Asia. We added three SNPs (rs2252214, rs4374177 and rs2239739) downstream to the four SNPs to construct the haplotype network again and found that --^SEA^ arose from the ααAAGCCAG haplotype which was only found in Asian populations (data not shown). Considering the geographical distribution in which the --^SEA^ has been found in only Thai, Filipino, Vietnamese, and Chinese populations [23], our phylogenetic network indicates that the --^SEA^ appears to come from an Asian-specific haplotypic background, is consistent with the localized distribution of this mutation, indicating a recent origin of the deletion.

The mechanism of protection in α^0^-thalassemia, and α^+^-thalassemia, remains unclear at the cellular level because there is no evidence for a reduced rate of invasion or growth of *P. falciparum* in red cells of the genotypes -α/αα, -α/-α, or --/αα. However, some reviews of the mechanisms of malaria resistance, based on *in vitro* work, have supplied some potential mechanisms [24,25]. The most relevant mechanism is an increased immune response against parasite-infected erythrocytes, which was supported by a case-control study carried out by Williams, et al [26]. Some other plausible explanation involves reduced rosetting, the amount of which correlates strongly with the severity of malaria infection. Another case-control study demonstrated that complement receptor 1 (CR1) expression required for rosette formation, is reduced on α-thalassemia red cells [25,27]. Recently, Fowkes et al. suggested that the increased erythrocyte count and microcytosis in children homozygous for α^+^-thalassemia may confer an advantage during acute infection with the malaria parasite *P. falciparum*[28]*.* This may provide an underlying mechanism for the --^SEA^ protection against malaria, considering that the phenotypic effect of α^0^-thalassemia heterozygotes appears to be the same as α^+^-thalassemia homozygotes. Moreover, many genetic variants against malaria are involved in hemoglobin-inherited disorders, erythrocyte polymorphisms, enzymopathies, and immunogenetic variants, which may be only a small proportion of the complex interaction between Plasmodium parasites and humans. By harvesting the fruit of high-throughput sequencing techniques and human genome analysis, we may identify some new loci with impacts on susceptibility to malaria by association studies. Such work may make the mechanism underlying the --^SEA^ and/or other α^0^-thalassemias clearer.

The fact that several hemoglobinopathies variants coexist in southern China make the expansion of the --^SEA^ more complex. These variants interact with each other. For example, the frequency of α-thalassaemias coexisting with β-thalassaemia could be higher than when it exits on its own because of the increased fitness of the α/β-thalassaemia heterozygote; α^0^-thalassemias and α^+^-thalassemias are in competition for the combination of these two alleles results in a more deleterious effects: HbH disease. According to the result of our previous epidemiological survey, the frequencies of the --^SEA^ and α^+^ deletion appear to be relatively similar [9,10]. Given the model proposed by Hedrick [22], --^SEA^ can persist at the current frequency for a long time, but will always eventually be outcompeted by α^+^. The interactions between the --^SEA^ and other variants mean that the spread of this allele is not a straightforward case of balancing selection. In Zhang’s study on β^CD41/42^, gene conversion was shown to play an important role in the spread of the 4 bp frame deletion [7]. Unfortunately, this process cannot be analogized to the --^SEA^ because the lengths of maximally converted tracts in gene conversion rarely exceed 1 kb [29]. Obviously, in addition to the role of natural selection, a more comprehensive mechanism of the spread of the --^SEA^ is deserving of further investigation.

## Conclusions

Our data support the hypothesis that the --^SEA^ allele has been subjected to recent balancing selection, triggered by malaria. It ensures the basis of functional experiments about mechanisms of protection in α^0^-thalassemia and α^+^-thalassemia. We plan to explore the mechanism underlying the --^SEA^ and/or other α^0^-thalassemias by some high-throughput sequencing techniques in our future work.

## Methods

### Sample

A total of 214 samples from Guangdong and Guangxi of southern China were collected in this study. They consisted of 76 --^SEA^/αα heterozygotes and 138 wild-type individuals. Genomic DNA was extracted by a standard phenol/chloroform method from leucocytes in peripheral blood. We recognized the limitation of our sampling strategy and thus built a constructed random sample (CRS) [30] on the basis of the population frequency of the --^SEA^ carrier by randomly excluding 65 --^SEA^/αα samples to arbitrarily maintain a total frequency of 7.4% (i.e. frequency of --^SEA^ allele was 3.7%) in the studied population. For those analyses in which random sample was preferred, such as LD analysis LRH test and network construction, we used CRS dataset rather than the whole dataset.All participants were recruited after providing written informed consent. The institutional review board of Southern Medical University approved this study.

### Gap-PCR to confirm heterozygotes

The Gap-PCR method was performed to discriminate the heterozygotes from normal individuals as reported by our laboratory [31]. In this assay, two primers were designed: one pair bridging the breakpoints producing a 740 bp fragment to identify the chromosomes with the --^SEA^ determinant, while the other was located in the deletion region to detect the normal chromosomes through a 1052 bp fragment.

### SNP selection

Because the α-globin gene cluster is located on chromosome 16p13.3, close to the telomere, we scanned a fragment from the beginning to 470 kb, about 300 kb behind the downstream deletion breakpoint, to screen appropriate SNPs. Data from HapMap release 27 were used. SNPs were selected based on their minor allele frequency (MAF ≥ 0.1) in the CHB population and haplotype-tagging SNPs were assessed using HAPLOVIEW 4.2 [32] under the parameter r^2^ ≥ 0.8. To reduce the genotyping cost, just one or two SNPs were picked out from each block or boundary. Additionally, three extra SNPs (rs77308790, rs2858925, rs3760053) in the proximal region upstream of the deletion breakpoint, from a study [unpublished] in our laboratory, were also included. As a result, a total of 28 SNPs encompassing the SEA deletion and covering a ~410 kb region were determined (Figure 1, Table 1).

### High resolution melting (HRM) analysis

A high-throughput technique termed high resolution melting of small amplicons (~40-90 bp) was used to genotype these markers after sequencing about 10 samples for each SNP. The common PCR system was 10 μl of 1 × PCR buffer (10 mM Tris-HCl pH 8.3, 50 mM KCL, 1.5 mM Mg^2+^), 30 ng of gDNA, 100 μM of each dNTP (Takara, Dalian, Liaoning, China), 0.3 μM of each primer, 0.4 U Taq polymerase (Takara, Dalian, China), 0.1 μM of each high- and low-temperature control [33], and 1 × LC Green Plus dye (Idaho Technology Inc., Utah, USA). Amplification was in a 96-well plate at 95°C for 5 min followed by 40-50 cycles (depend on the assay) at 95°C for 30 sec, 56-65°C (depending on the assay) for 30 s, 72°C for 30 s, with a denaturation at 95°C for 30 s after the thermal cycling and cooling to 10°C for 1 min. After amplification, HRM analysis was performed on a Light Scanner System (Idaho Technology Inc., Utah, USA) from 55°C to 95°C at 0.1 °C/s and the data were analyzed with the Light Scanner System 2.0 Software program (Idaho Technology). A list of the primers and corresponding SNP identifiers is shown in Additional file 1: Table S1.

### Hardy – Weinberg equilibrium tests

In the CRS dataset, we performed a Hardy-Weinberg equilibrium test. None of the markers, including the --^SEA^ allele, deviated from HWE at a significance level of 0.05 except rs216091 (P = 0.05). A double check, sequencing 22 individuals for rs216091, ruled out the possibility of genotyping errors. Additionally, the genotype frequencies of rs216091 were similar to that of CHS population, which is believed to represent the Southern Chinese population, in the SGVP (Singapore Genome Variation Project) [34].

### Data analysis

Haplotypes were inferred by a Bayesian statistical method with the PHASE 2.1 software [35] using the default parameter set with 1,000 iterations. This program assigns a pair of haplotypes for each diploid entry and provides probability scores for each uncertain position. Reconstructed haplotypes were inserted into Haploview v. 4.2 to show LD pattern and |D’| value which is a measurement of the deviation of the observed frequency of a haplotype from the expected. To evaluate whether the long-range LD around the focal allele was due to large-scale deletion, we compared the minimum number of recombination events, namely RM, between haplotypes with the --^SEA^ allele and wild-type in our samples using a bootstrap approach. The distribution of difference of RM was obtained in three steps: (1) 428 chromosomes were randomly divided into two groups. One contained 76 and the other contained 352 chromosomes. (2) The difference of RM between the two groups was calculated. (3) Steps 1-2 were repeated 5000 times from which we obtained the distribution of the statistics in which we were interested. Relative extended haplotype homozygosity (REHH) values were calculated using the Sweep 1.1 software (http://www.broadinstitute.org/mpg/sweep/). REHH values of the core haplotypes with frequencies similar (3.1% < MAF < 4.2%) to the --^SEA^ on chromosome 16 were obtained for CHS and calculated as empirical data. The inferred haplotype network was constructed by a median-joining method with NETWORK 4.6.1.0 [36] using the default settings.

## Competing interests

The authors declare that they have no competing interest.

## Authors’ contributions

QWQ designed the research, performed the lab experiments and data analyses, and wrote the manuscript. DDW provided support for the study, contributed to the analyses and revised the manuscript. LHY performed the lab experiments and data analyses. TZY and YHL collected clinical data and provided clinical support. WZ and YPZ revised the manuscript and contributed to discussion. ZTL performed the experiments and produced experimental data. XMX and TZY conceived the study and revised the manuscript. All authors read and approved the final manuscript.

## Supplementary Material

Additional file 1 **List of the 28 SNPs, their indentifiers, the primers used for prior-HRM amplification of them.** The sequences are shown from 5′ to 3′ end. Click here for file
