# Supplementary material for: Evidence of recent natural selection on the Southeast Asian deletion (--SEA) causing α-thalassemia in South China
Source: BMC Evol Biol. 2013 Mar 11;13:63. doi: 10.1186/1471-2148-13-63 (PMC3626844; doi:10.1186/1471-2148-13-63)
Supplement: Additional file 1 — List of the 28 SNPs, their indentifiers, the primers used for prior-HRM amplification of them. The sequences are shown from 5′ to 3′ end. [file 1471-2148-13-63-S1.doc]

**Table S1**. List of the 28 SNPs, their indentifiers, the primers used for prior-HRM amplification of them. The sequences are shown from 5’ to 3’ end.

| **SNP rs #** | **Amplicon Size (bp)** | Forward Oligo Sequence | Reverse Oligo Sequence |
| --- | --- | --- | --- |
| rs2541593 | 46 | GGCGATGGAGCAGCAGT | CGACTCTCGCAGGCCTT |
| rs216606 | 89 | GGGCCACCTCTGAGTCT | AAGCCCCTTGCCGAGCA |
| rs3785288 | 67 | TTGTGGGGTGGGATTGT | GCCACTTGAAGGTTTATCTGGG |
| rs2541622 | 78 | CACAGGGCACTTCACAA | CCCTGAAGAATGTGCAG |
| rs216091 | 49 | GGTCAGGAAAGAATGCTA | GAAAAAGGCTCCTAAGC |
| rs2238368 | 53 | TAGAAGAGACCACTGAGGAC | GAAGCATGACCAGGTTTG |
| rs2562164 | 67 | AATACCCAGGGAACACT | GGATATTGTCCAGTGTCTC |
| rs2562185 | 60 | CAAAAGGTTTGTGAGTTCTCC | AGTCTCATTTCCATCCCAGA |
| rs77308790 | 55 | TCACGGAGGCATCTCTC | GATTAGTGGTTGTCAGGGC |
| rs6600143 | 73 | ATATCTGACAAGGGATTGC | GGGTGGTTTATTCTCTTAAGG |
| rs2858925 | 44 | CCAACGCGGGCAGATTA | GGTTAGTCTGGAACTCCTG |
| rs3760053 | 65 | CTACCCTGACTCTGGCT | CAGGAGACTGACACCCT |
| rs1211375 | 88 | GCTTGCTGCTTTACTGACA | GGACCTAGCCATTGGAGAT |
| rs3918352 | 84 | CAGGGGGAGGCTGGCTAATT | TCTCCCTTCACACTGATGGAGC |
| rs1203974 | 69 | CTGTGTTCTCCCCATCAA | TGTGTATTCCACTCCAGTTG |
| rs11248914 | 61 | TCTTTGGACCTGTCTCATG | GCCAGRCAGTTCATGATTC |
| rs2252214 | 68 | CCTGGCCTTATTAATGATT | AACACAGACTGATCTAAGA |
| rs4374177 | 59 | AGTGGAATTGCTGAACCCT | GCTTGTCAGCTCCTCAGA |
| rs2239739 | 57 | TAACCGTGCAGTGTGGA | ACACGCTGACATCAGAGA |
| rs9940585 | 65 | TGGACCCTGCTCCAGCACATC | CATCCCCACCTCATCTCGTCT |
| rs2685126 | 57 | TGGTCCCACCTTCTCATA | GCCAAGAAGAACATCCGA |
| rs214247 | 70 | CAAGTTAACAGGTGCTGAA | GAGATGCAACTTGGAAT |
| rs1981492 | 74 | TGTTCCACAGTAGACGAGG | TACTCCACGTTCCTTGAAGT |
| rs11648673 | 71 | CAAAGCGGAAATGACAG | CTCACCCAAGCACTGTT |
| rs1573733 | 70 | GTGGCATCACAGAAGACATGAG | TCACAGCACTGCACTCCA |
| rs4984666 | 84 | CATGCTGTGCTATCTACC | CCACATTCATAGTTGCAGG |
| rs1698232 | 86 | CATCACACTTACAACCAG | CACTCATATCTCCCAACAC |
| rs3785301 | 87 | GGGCGGTGAGCTAGTCATC | TGCAGCCACCTCCACCA |
